# The relationship between renin-angiotensin system-modulating medication and depression: a systematic review

**DOI:** 10.1038/s41398-026-04176-2

**Published:** 2026-06-26

**Authors:** Sanika Kulkarni, Divya Prasad, Sarah Bauermeister, Andrea Reinecke

**Affiliations:** Department of Psychiatry, University of Oxford, Oxford, UK

**Keywords:** Depression, Clinical pharmacology

## Abstract

**Introduction:**

The renin-angiotensin system (RAS) is implicated in stress, inflammation, and reward-related neurobiology, and RAS inhibition shows antidepressant-like effects in preclinical models. Whether angiotensin-converting enzyme inhibitors (ACEIs) and angiotensin receptor blockers (ARBs) are associated with depression outcomes in adults remains uncertain.

**Methods:**

We searched MEDLINE, Embase, PsycINFO, APA PsycArticles, and AMED from inception to November 2024 for observational studies examining ACEI or ARB exposure and depression outcomes in adults. Risk of bias was assessed with the Quality in Prognosis Studies (QUIPS) tool. Due to methodological heterogeneity, no meta-analysis was performed, and findings were synthesised using structured direction of effect methods, with sensitivity synthesis restricted to studies at low risk of confounding.

**Results:**

Fifteen studies involving more than 7 million participants were included. Eight studies reported protective associations between ACEI or ARB exposure and depression outcomes, six found no clear association, and one linked data cohort reported a modest increase in major depressive disorder with angiotensin antagonist monotherapy compared with patients not receiving antihypertensive treatment. Protective associations were more common in large registry cohorts using clinician-coded outcomes, whereas studies using symptom scales or prescription-based proxies were more often null. Among studies with stronger confounding control, findings were mixed.

**Conclusion:**

Observational evidence suggests a modest protective association between ACEI or ARB exposure and depression outcomes. However, heterogeneity in exposure and outcome definitions, together with residual confounding, limits causal inference. Prospective studies using bias-resistant designs and improved characterisation of incident depression, and better characterisation of drug-specific exposure are needed.

## Introduction

Depression is a major contributor to the global burden of disease, affecting more than 280 million individuals worldwide. It is strongly associated with reduced quality of life and functioning, increased health care utilisation, and premature mortality [1, 2]. Despite the availability of evidence-based pharmacological and psychological therapies, treatment of depression remains a significant challenge in clinical practice. Approximately 30–50% of patients do not respond to first-line antidepressants, and relapse rates after remission are substantial [3, 4]. These limitations underscore the need for novel therapeutic strategies that go beyond the current therapeutic pathways.

In recent years, there has been growing interest in the potential role of the renin-angiotensin system (RAS) in the modulation of mood and depression. The RAS is a critical hormonal cascade that regulates blood pressure, fluid homeostasis, and cardiovascular remodeling. Its principal effector, angiotensin II, is generated by angiotensin-converting enzyme (ACE) and acts on angiotensin type-1 (AT1) receptors to induce vasoconstriction, aldosterone release, sympathetic nervous system activation, oxidative stress, and inflammation [5]. A second receptor type, the angiotensin type-2 (AT2) receptor, mediates generally opposing, counter-regulatory effects, including vasodilation, anti-proliferative signaling, and anti-inflammatory responses [6].

However, beyond the well-known peripheral RAS, a local brain RAS has been identified. RAS receptors are expressed in brain regions implicated in stress and emotion regulation, such as the hypothalamus, amygdala, and hippocampus [7]. Consistent with this neuroanatomical overlap, preclinical studies have linked hyperactivation of RAS pathways to depression-related neurobiology. For instance, acute stress in rodents elevates brain angiotensin II levels, and central administration of angiotensin II induces depressive-like behaviours [7, 8]. Conversely, pharmacologically blocking the RAS has been suggested to yield antidepressant-like effects in animal models. These agents, widely used as antihypertensive drugs, include ACE inhibitors (ACEIs) which interfere with the production of angiotensin II and AT1 receptor blockers (ARBs) which prevent angiotensin II from binding to its main receptor (AT₁). The systemic and central administration of these antihypertensives has been shown to rapidly reduce behavioural markers of depression, and to enhance neurogenesis in brain regions implicated in emotional regulation [9, 10].

In humans, recent experimental studies in healthy volunteers suggest mechanistic effects of the ARB losartan opposite to those seen in depression: a single dose of the medication compared to placebo reduced learning from aversive outcomes, increased bias towards positive over negative information [11] and increased response to rewarding outcomes in the striatum, the reward-related circuitry of the brain [12]. Such effects are opposite to those typically seen in depression [13, 14], and they are similar to mechanistic effects seen during remission from depression [15]. These findings provide preliminary evidence that the RAS may play an important yet so far under-researched role in mood and depression in humans.

A number of observational studies over the past two decades have explored associations between various antihypertensive drugs and mood disorders. However, relatively few analyses have focused specifically on RAS-modulating antihypertensives (i.e. ACEIs and ARBs). The evidence to date is suggestive but inconsistent. Some large-scale studies have reported that patients on RAS inhibitors tend to show lower rates of depression compared to those on other blood pressure medications [16]. Similarly, a nationwide study in Denmark found that several specific antihypertensive agents, including but not limited to RAS-modulating drugs (two ACE inhibitors), were linked to a modestly reduced risk of developing depression. Importantly, no antihypertensive was linked to an increased risk [17]. Other studies have yielded conflicting results [18–20], suggesting no association between RAS drugs and depression outcomes. To illustrate, a cross-sectional analysis reported no significant difference in depressive symptom prevalence between users of ACEIs/ARBs and users of other antihypertensives, while noting a higher odds ratio of depression in those taking beta blockers [21]. Discrepancies between studies likely stem from variability in design and methodology, including differences in population demographics, follow-up duration, and how depression was defined (clinical diagnosis, self-reported symptoms or antidepressant prescription). Variation in outcome measures and potential confounding factors, such as underlying cardiovascular disease severity or indication bias, further limits the current conclusions. Taken together, the existing literature provides intriguing but inconclusive evidence regarding the relationship between RAS-modulating medication and depression.

Given the variability and limitations in the existing literature, a focused synthesis of observational evidence is needed to further determine whether use of ACEIs or ARBs is associated with depression outcomes in real-world populations. The present work aims to systematically review observational studies that specifically evaluate the association between ACEIs/ARBs and depression-related outcomes. This analysis will account for differences in study design, exposure and outcome definitions, and methodological quality, and it will synthesise findings using structured direction-of-effect methods. Although other antihypertensive classes are referenced for context, our primary focus is on ACEIs and ARBs. This analysis will inform future investigations into the role of the RAS in depression, and it may guide decisions for individuals with comorbid cardiovascular and psychiatric risk profiles.

## Methods

### Protocol and registration

This systematic review was conducted in accordance with the Preferred Reporting Items for Systematic Reviews and Meta-Analyses (PRISMA) 2020 guidelines [22]. The review protocol was prospectively registered on PROSPERO (ID: CRD42024614664).

### Eligibility criteria

We included studies which were peer-reviewed and published in English, and were observational studies (cohort, case-control, or cross-sectional) that evaluated the association between antihypertensive medication use and depression outcomes in adults (aged ≥18 years). Eligible studies had to examine at least one RAS-modulating antihypertensive drug (ACEIs or ARBs) and report depression as an outcome, defined by clinical diagnosis (e.g., ICD-codes, validated self-report scales (e.g., Patient Health Questionnaire-9 (PHQ-9) [23], Center for Epidemiologic Studies Depression Scale (CES-D) [24], Beck’s Depression Inventory (BDI) [25], Structured Clinical Interview for DSM-IV, non-Patient Edition (SCID-I/NP) [26]), or antidepressant prescription records.

Studies were excluded if they met any of the following criteria:

- Focused on non-human subjects or exclusively on paediatric populations.
- Were review articles, meta-analyses, conference abstracts, editorials, or book chapters (i.e. not original research).
- Examined only genetic or molecular components of the RAS without any pharmacological RAS inhibitor exposure.
- Did not report any depression-related outcome.

### Information sources and search strategy

A comprehensive literature search was performed across multiple databases from inception through November 2024. We searched five electronic databases via the Ovid platform: MEDLINE, Embase, PsycINFO, APA PsycArticles, and AMED. Keywords and controlled vocabulary terms were used to identify relevant literature, including:

- Antihypertensives: “ACE inhibitors”, “angiotensin receptor blockers”, “renin–angiotensin system”, “antihypertensive agents”
- Depression: “depression”, “major depressive disorder”, “depressive symptoms”, “mood disorder” Boolean operators (AND/OR) were used to combine terms.

We also searched PubMed and Google Scholar to identify any additional relevant publications, including grey literature or recent in-press articles that might not yet be indexed in the primary databases. In addition, reference lists of all included studies were hand-searched to capture any studies that the database searches might have missed.

### Study selection

**Fig. 1:**
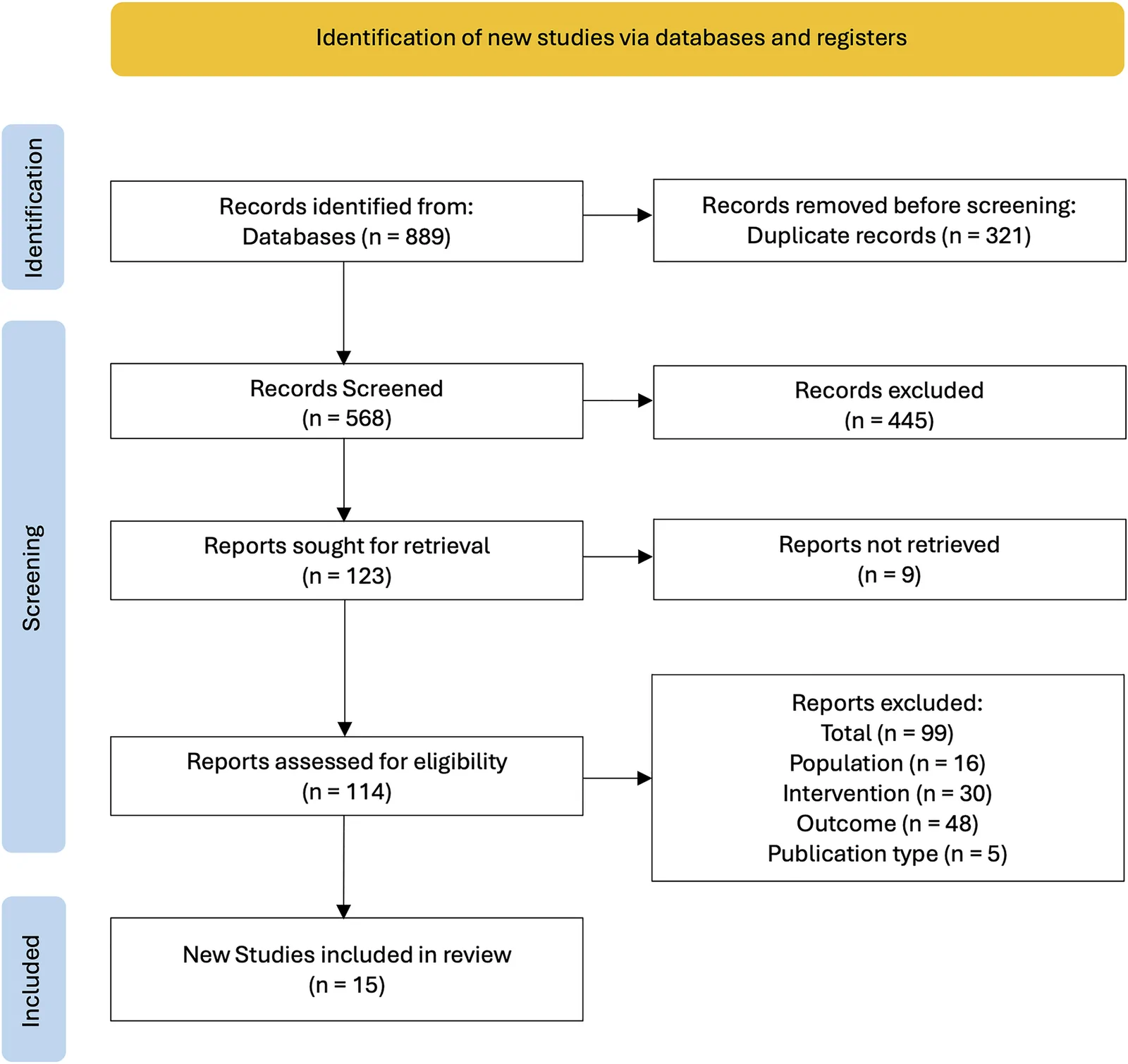
PRISMA flow diagram of the screening process of the review.

All search results were imported into Rayyan, a web-based application for screening references [27]. After removing duplicate records, two reviewers independently screened titles and abstracts for potential relevance. Studies marked for inclusion by either reviewer underwent full-text assessment. A third reviewer resolved any conflicts at this stage. For full-text screening, two reviewers independently evaluated each article against the eligibility criteria. Any disagreements at either the title/abstract or full-text screening stage were resolved through discussion. The third reviewer was available to adjudicate conflicts and helped achieve consensus when needed. Final inclusion decisions were confirmed in consultation with a senior supervisor to ensure consistency and rigour in applying the criteria. A PRISMA flow diagram (Fig. 1) summarises the screening process.

### Data extraction

Data from each study were extracted using a standardized form by two reviewers working independently. The following information was collected from each included study:

- **Study characteristics:** author(s), publication year, country, study design, and sample size.
- **Participant characteristics:** key demographics (e.g., mean age) and clinical setting or population (e.g., community, primary care, hypertensive patients).
- **Exposure details:** antihypertensive class and, where reported, specific agent; source of exposure ascertainment (prescribing, dispensing, or self-report); whether the study used a new user or prevalent user design; and availability of dose, duration, adherence, or blood-brain barrier penetration information.
- **Outcome measures:** definition of depression outcome and how it was assessed (clinical diagnostic criteria, specific screening or symptom scale, antidepressant use, and timing of outcome assessment).
- **Covariates:** confounding variables accounted for in statistical analyses (e.g., age, sex, comorbid conditions, other medications, and any baseline mental health measures).
- **Effect estimates:** the reported association between RAS-modulating drug use and depression outcome, typically expressed as adjusted odds ratios (ORs), relative risks (RRs), or hazard ratios (HRs) with corresponding 95% confidence intervals (CIs).

The two reviewers compared their extracted data and resolved any discrepancies by consensus. If necessary, a third reviewer was consulted to settle outstanding differences and a senior supervisor provided guidance at every step.

### Risk of bias assessment

The risk of bias in each included study was evaluated using the Quality in Prognosis Studies (QUIPS) tool [28]. This tool assesses potential bias across six domains relevant to prognostic and observational studies: (1) study participation, (2) study attrition, (3) prognostic factor (exposure) measurement, (4) outcome measurement, (5) study confounding, and (6) statistical analysis and reporting. For each domain, each study was rated as having a low, moderate, or high risk of bias based on the criteria outlined by QUIPS. Three reviewers independently appraised every included study across all six domains. The reviewers compared assessments and discussed any differences; a final judgment for each domain was reached by consensus to ensure consistency in quality ratings. Studies were not excluded based on risk of bias, but these assessments informed the interpretation of findings.

### Data synthesis

Given substantial clinical and methodological heterogeneity in study designs, exposure ascertainment, comparator choice, and depression outcome definitions, statistical pooling was not considered appropriate. We therefore used structured synthesis methods based on direction of effect, following current guidance for synthesis without meta-analysis [29]. For each study, we classified the most adjusted estimate for the primary depression outcome as indicating a protective association (point estimate below 1.0), a harmful association (point estimate above 1.0), or no clear association (confidence interval crossing 1.0). Where multiple estimates were reported, we prioritised class-specific results (ACEI and ARB separately where available) and outcomes reflecting incident depression during follow-up. For continuous symptom-scale outcomes, direction was defined relative to the null value of 0, with lower depressive symptom severity or greater improvement in the exposed group classified as protective, and higher symptom severity classified as harmful.

Findings were summarised using (a) an effect direction plot to visualise direction of association across studies alongside key study characteristics and risk of bias, and (b) vote counting based on direction of effect, stratified by study design and risk of bias. As a robustness check, we conducted a sensitivity synthesis, restricting interpretation to studies judged as having a low risk of bias in the QUIPS confounding domain and to bias-resistant designs where available.

## Results

### Study selection and characteristics

The systematic search identified 889 records. After removal of duplicates, 568 titles and abstracts were screened, and a total of 114 full-text articles were sought for review in detail. Of these, 99 were excluded for reasons such as ineligible design, outcomes not related to depression, exposures not including ACEIs or ARBs, or insufficient data. Ultimately, 15 studies met inclusion criteria and were included in this review. The study selection process is illustrated in the PRISMA flow diagram in Fig. 1.

The 15 studies included in this review were published between 1996 and 2024, with a combined sample size of more than 7 million participants (Tables 1 and 2). Most of these adults used RAS medication for the treatment of hypertension, although some studies included other primary indications such as diabetes, cardiovascular disease or chronic kidney disease [16, 19, 30]. Study designs included large registry-based cohorts (*N* = 5), retrospective or observational cohort studies (*N* = 5), cross-sectional analyses (*N* = 4), and one small prospective comparative cohort (*N* = 1). Registry and cohort studies typically drew upon national health registries, insurance claims, electronic health records, or clinical diagnoses, whereas cross-sectional and clinical studies evaluated patients in community or healthcare settings.

**Table 1:** **(A and B): Summary of included studies examining RAS-modulating drugs and depression outcomes**, including studies that found protective effects between RAS drugs and depression outcome (A), and studies that did not find protective effects between RAS drugs and depression (B). Both sections with studies in chronological order of the year of publishing.

Study	Year	Design	Sample size	Population		Exposure	Comparison	Effect Measure	Depression outcome measure	Key findings
**A: Studies that found protective effects between RAS drugs and depression**										
**Braszko et al**. [31]	2003	Prospective comparative cohort	54 (39 hypertensive patients + 15 normotensive controls)	Hypertensive outpatients		ACEIs (captopril, enalapril)	Untreated hypertensives, and normotensive	Both captopril (7.50 ± 1.31) and enalapril (8.89 ± 1.75) significantly reduced symptoms vs untreated	Self-report scale (BDI)	ACEI treatment associated with improved mood and cognition compared with untreated
**Nasr et al**. [34]	2011	Cross-sectional	378	Hypertensive adults		ACEIs, ARBs	Other classes / non-use	Proportional differences / chi-square statistics χ² = 8.88, *p* = 0.003	Prescription records (antidepressant use, proxy)	ACEI/ARB users reported lower antidepressant use (proxy for depression outcome)
**Boal et al**. [16]	2016	Nationwide cohort (data from 1980 to 2013)	144,066	Adults on antihypertensive monotherapy		ACEIs, ARBs, beta-blockers, CCBs, thiazides	Between classes	Beta-blockers vs others: HR = 2.11 (95% CI 1.12–3.98, *p* < 0.05)	ICD-9 before 1996, ICD-10 after 1996	Lowest risk with ACEIs/ARBs; higher risk with beta-blockers and CCBs compared with ACEIs/ARBs
**Williams et al**. [32]	2016	Retrospective cohort (Nested case-control)	961	Older adults with hypertension		ACEIs	Non-use / other classes	OR = 0.15 (95% CI: 0.04-0.51, *p* = 0.003)	Structured Clinical Interview for DSM-IV, non-patient edition (SCID-I/NP)	ACEIs associated with a significant protective effect against developing depression
**Cao et al**. [35]	2019	Retrospective cohort	181,709	Hypertensive patients		ACEIs, ARBs, beta-blockers, CCBs, diuretics	Between classes	Incidence density: ARB 2.30 (95% CI 2.16–2.43) - lowest, ACEI 3.10 (2.91–3.29)	Clinical diagnosis (ICD-10) and Prescription records (antidepressant use, proxy)	ARB users showed the lowest incidence of depression (57% lower hazard) compared with other classes
**Kessing et al**. [17]	2020	Nationwide registry cohort	5.4 million (3.7 million on antihypertensive)	Adults in Denmark		ACEI/ARBs, beta-blockers	Diuretics	Angiotensin agents: HR ~ 0.91–0.97, decreased risk	Clinical diagnosis (ICD-10, registry) and prescription data	Continued use of ACEIs/ARBs, CCBs, and beta-blockers associated with lower depression rates; no effect for diuretics
**Colbourne et al**. [36]	2021	EHR network cohort (TriNetX)	58.6 million database ( ~ 2 million from the total cohorts analysed)	General population (international)		Antihypertensive classes	Between classes	Depression RR ≈ 0.93–0.94 (95% CI 0.92–0.96) favouring ARBs	Clinical diagnosis (ICD-10)	ARBs associated with lower risk of depression and affective disorders(protective effect), ACEIs showing slightly protective association
**Lee et al**. [33]	2023	Nationwide cohort	61,090	Newly diagnosed hypertensive patients		ACEIs, ARBs, CCBs	Between classes	ACEI + CCB vs CCB-only: HR 0.87 (95% CI 0.77–0.99), protective	Clinical diagnosis (ICD-10, registry)	ACEIs associated with decreased risk of depression; ARBs did not show similar reduction
**B: Included studies that did not find protective effects between RAS drugs and depression**										
**Gerstman et al**. [18]	1996	Retrospective cohort	~50,000	US insurance database, antihypertensive users	ACEIs, beta-blockers, CCBs, diuretics		Between classes	Beta-blockers vs non beta-blockers RR 0.8 (95% CI: 0.3–1.9)	Clinical diagnosis (ICD-9, insurance claims)	Overall depression rates low across all classes; no increased risk of depression with beta-blockers
**Simonson et al**. [20]	2011	Cross-sectional	5000+	US nursing home residents	Antihypertensive classes		Between classes	Any antihypertensive: OR 0.76 (95% CI 0.63–0.90) → lower odds of depression, not specific to RAS drugs	Clinical diagnosis/treatment (chart review, claims)	Antihypertensives overall showed reduced likelihood of depression, but not specifically ACEI/ARBs
**Michal et al**. [38]	2013	Cross-sectional	5000	Hypertensive adults	Antihypertensives (not separated)		Normotensive participants	ACE inhibitors: OR 1.23 (95% CI 0.91–1.66), nonsignificant, but positive trend	Self-report scale (PHQ-9)	Beta-blockers and ACEI correlated with increased somatic depressive symptoms
**Agustini et al**. [21]	2020	Cross-sectional	~17,000 pooled	Older adults with hypertension	ACEIs, ARBs, beta-blockers, CCBs		Between classes	ARB, CCB vs other classes: No significant association (OR ≈ 1.0–1.08; all ns)	Self-report scale (CES-D)	ACEI/ARB not significantly associated with depression, beta-blockers showed high risk
**Shaw et al**. [19]	2021	Nationwide linked-data cohort	~1.8 million	Adults with antihypertensive exposure	All classes incl. angiotensin antagonists		Between classes	Depression risk AAs: HR 1.17 (95% CI 1.04–1.31)	New-onset major depressive disorder (ICD-10, registry); bipolar disorder separately assessed	No protective effect of antihypertensives; possible protective signal for bipolar disorder with angiotensin drugs
**van Sloten et al**. [30]	2022	New-user PS-matched cohort	12,938 pairs (~25,876)	Older adults in England (CPRD)	ACEI/ARB initiators		Thiazide initiators, diuretics, CCBs	HR 0.96 (0.79–1.15), null	Clinical diagnosis + prescription, history of self-harm	No lower risk of depression with ACEI/ARB vs thiazides (null association)
**Gammoh et al**. [37]	2024	Cross-sectional	431	Hypertensive clinic patients (Jordan)	ACEIs/ARBs, beta-blockers, diuretics, CCBs		Other classes / non-use	No significant associations	Self-report scale (PHQ-9)	No individual antihypertensive was associated with severe depression

**Table 2:** **(A and B):** Risk of Bias Summary (using the Quality in Prognosis Studies (QUIPS) Tool Assessment), including studies that found protective effects between RAS drugs and depression (A), and studies that did not find protective effects between RAS drugs and depression (B).

Study	Study Participation	Study Attrition	Prognostic factor Measurement	Outcome Measurement	Study Confounding	Statistical Analysis & Reporting
**A: Risk of Bias Summary - QUIPS tool with included studies that found protective effects between RAS drugs and depression**						
Braszko et al., [31]	Low	Moderate	Low	Low	Moderate	Moderate
Nasr et al., [34]	Low	Moderate	Moderate	Moderate	High	Moderate
Boal et al., [16]	Low	Moderate	Moderate	Low	Moderate	Low
Williams et al., [32]	Moderate	Moderate	Moderate	Low	Moderate	Low
Cao et al., [35]	Low	Moderate	Moderate	Low	Moderate	Low
Kessing et al., [17]	Low	Low	Moderate	Low	Low	Low
Colbourne et al., [36]	Low	Moderate	Moderate	Low	Moderate	Low
Lee et al., [33]	Low	Moderate	Moderate	Moderate	Moderate	Moderate
**B: Risk of Bias Summary - QUIPS tool with included studies that did not find protective effects between RAS drugs and depression**						
Gerstman et al., [18]	Low	Moderate	Moderate	Low	Moderate	Low
Simonson et al., [20]	Moderate	Moderate	Moderate	Low	High	Moderate
Michal et al., [38]	Low	Moderate	Moderate	Moderate	Moderate	Moderate
Agustini et al., [21]	Low	Moderate	Moderate	Low	Moderate	Low
Shaw et al., [19]	Low	Moderate	Moderate	Low	Moderate	Low
van Sloten et al., [30]	Low	Low	Moderate	Low	Low	Low
Gammoh et al., [37]	Moderate	Moderate	Moderate	Moderate	Moderate	Moderate

Geographically, the evidence base spanned Europe, Asia, North America, and Australia. Large registry-based studies were conducted in Denmark, Scotland, Korea, the Netherlands, and the United States, with additional retrospective cohorts from China, the UK, and Australia. Smaller cross-sectional studies came from Jordan, Germany, and the US, with one prospective comparative cohort from Poland.

Exposure definitions varied across studies. Some analysed ACEIs and ARBs as separate drug classes, while others grouped them as angiotensin agents. Several restricted analyses to monotherapy cohorts to minimise confounding from combination therapy. In most cases, exposure was defined by prescription or dispensing records, whereas some cross-sectional studies relied on patient self-report. Comparison groups included other antihypertensive classes such as β-blockers, CCBs, and thiazide diuretics. In a few cases, untreated hypertensive patients or those receiving mixed therapy served as comparators.

The outcomes of the studies were heterogeneous. Out of the fifteen, there were seven studies that defined depression through ICD-coded (ICD-9 or ICD-10) clinical diagnoses, two studies used antidepressant prescriptions as a proxy, four relied on validated self-reported symptom scales (e.g., PHQ, CES-D, etc.), and two examined broader psychiatric disorder onset or recurrence. The extracted data are summarised in Table 1, with Table 1A presenting studies suggesting protective associations and Table 1B presenting studies not showing protective associations.

### Findings by drug class

#### Overall findings

Across the fifteen included studies, direction of association between ACEI or ARB exposure and depression outcomes varied. Eight studies indicated protective associations with depression outcomes, six reported no clear association, and one large linked-data cohort reported a modest increased risk of new-onset major depressive disorder with angiotensin antagonist monotherapy when compared with no antihypertensive treatment. Effect estimates were generally small and depression outcomes and exposure definitions varied widely across studies, limiting causal inference.

#### Structured synthesis

**Fig. 2:**
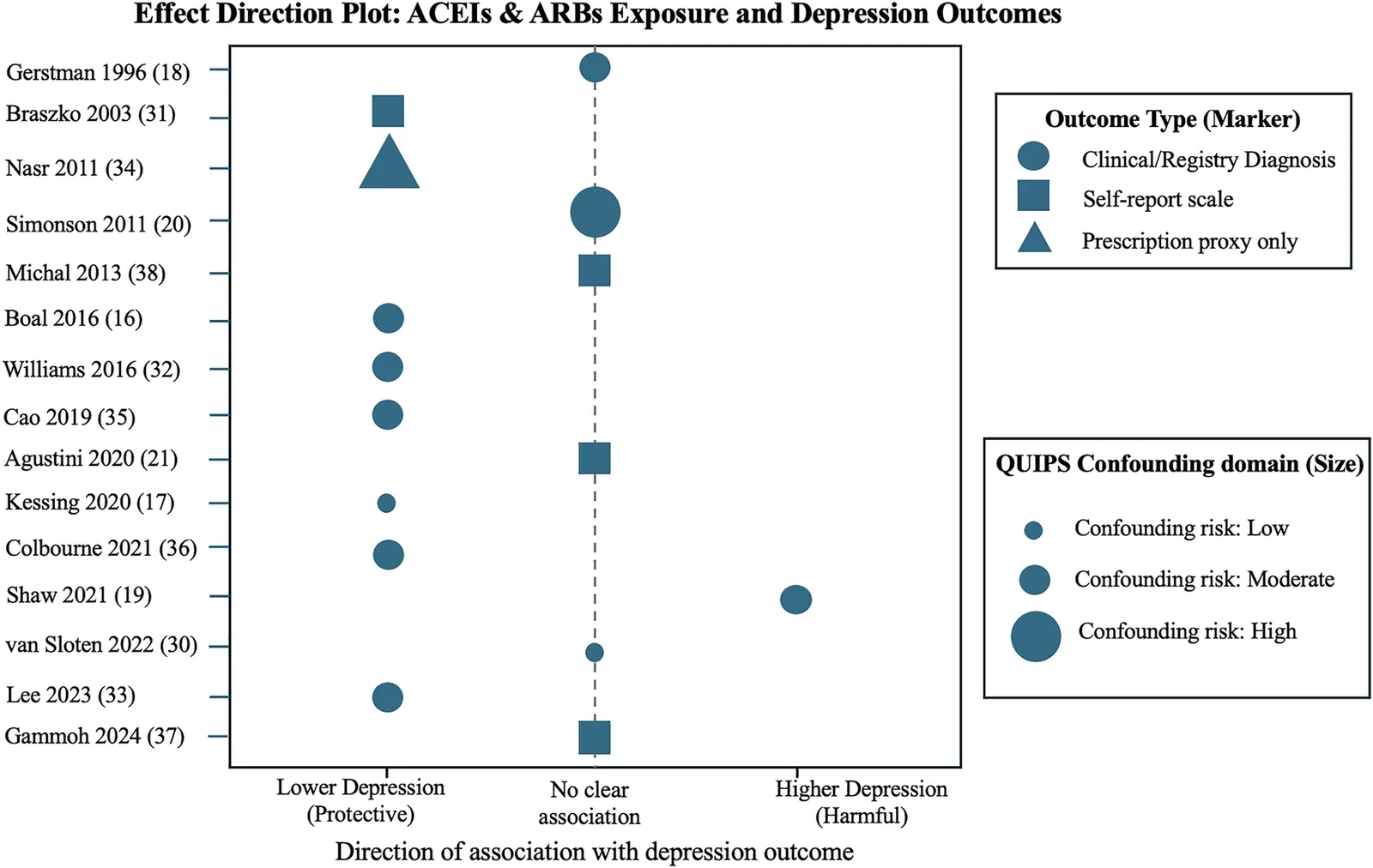
Effect direction plot summarising the direction of association between ACEI or ARB exposure and depression outcomes across included studies. Each study is positioned according to whether the primary comparative estimate indicated lower depression risk (protective), no clear association (confidence interval crossing the null), or higher depression risk (harm). Marker shape denotes outcome type and marker size denotes risk of bias in the QUIPS confounding domain.

In vote counting based on direction of effect, protective associations were more common in registry or electronic health record cohorts using clinician-coded depression outcomes, whereas cross-sectional studies using symptom scales were usually null. In sensitivity synthesis restricted to the two studies rated at lower risk in the QUIPS confounding domain, one study [17] suggested a modest protective association, whereas the other study [30], using a new-user active-comparator propensity-score-matched design, found no clear association; the more bias-resistant evidence therefore did not consistently support a protective effect. The effect of direction plot is shown in Fig. 2.

#### ACE inhibitors (ACEIs)

In several of the included studies, evidence suggested a protective association for ACEIs, but its strength varied materially by design and endpoint. A prospective cohort study found that the ACEI captopril and enalapril improved mood symptoms in hypertensive patients [31]. Another cohort study observed reduced risk of mood disorder diagnoses in ACEI users (OR = 0.15; 95% CI 0.04–0.51, *p* = 0.003), although that estimate was derived from a smaller sample and should be interpreted cautiously [32]. In larger cohorts, enalapril and ramipril were among agents reported to be associated with lower depression incidence [17], and ACEI initiation was identified as protective against major depressive disorder in a nationwide Korean cohort [33]. At the same time, class-specific inference remains constrained because some supportive evidence came from mixed ACEI and ARB exposure definitions or prescription-based proxies rather than incident depressive disorder itself [34, 35].

#### Angiotensin receptor blockers (ARBs)

Evidence supporting a protective association for ARBs mainly came from larger healthcare datasets. A large cohort study showed that ARB users had the lowest incidence of depression compared with other antihypertensive classes in a large Chinese cohort [35]. Moreover, in other large cohort studies, it was suggested that ARB monotherapy was associated with reduced hospital admissions for mood disorders compared with other classes [16], and also observations of a reduced risk of depression (RR ~ 0.93–0.94, 95% CI 0.92–0.96) with ARB exposure [36]. However, the new-user propensity-score-matched cohort found no clear reduction in incident depression [30]. In addition, several studies grouped ARBs with ACEIs or did not distinguish individual agents, making it difficult to determine whether the observed pattern reflects a class effect, particular compounds, or residual confounding. The ARB evidence is therefore suggestive, but it remains insufficient to support a consistent class-specific protective interpretation.

#### Null and adverse associations

Six of the fifteen studies reported no clear association between ACEI or ARB exposure and depression outcomes, and one large linked-data cohort reported a modest increase in new-onset major depressive disorder with angiotensin antagonist monotherapy compared with matched patients not receiving antihypertensive treatment. These findings arose across more than one design. Null results were reported in cross-sectional studies using self-reported symptom scales [21, 37, 38], but also in the new-user propensity-score-matched cohort [30], which offers stronger control of confounding by indication than broader prevalent-user or comparisons with patients not receiving antihypertensive therapy. Several large registry-based studies also did not identify a clear protective association [18–20]. These findings suggest that any protective signal is sensitive to study design, comparator choice, and outcome definition.

#### Risk of bias

Using the QUIPS tool, most studies were rated as having low to moderate risk of bias (as seen below in Table 2). The most frequent concern was residual confounding, as many studies did not fully adjust for prior psychiatric history, socioeconomic factors, or healthcare utilisation. There was heightened potential for outcome misclassification in studies using antidepressant prescriptions as a proxy for depression, because antidepressants may be prescribed for non-depressive indications. Potential exposure misclassification was possible where prescribing data were used without information on adherence, dose, or duration. Selection bias appeared limited in large registry-based cohorts but was more plausible in smaller clinical studies with potential participation bias. Statistical analysis and reporting were generally strong, with most studies using multivariable adjustment and a minority employing propensity-score methods.

## Discussion

This systematic review evaluated observational evidence on the relationship between RAS-modulating antihypertensive drugs and depression outcomes in adults. While previous studies have examined broader associations between antihypertensive use and mood disorders, this review focuses specifically on RAS-modulating agents and incorporates structured synthesis to interpret a heterogeneous evidence base.

Across fifteen studies, the evidence pointed toward a modest protective association between ACEI or ARB exposure and depression outcomes, mainly in large registry and electronic health record cohorts. However, this evidence is inconsistent in analyses with stronger confounding control and in studies using symptom-based or proxy outcomes, limiting causal inference.

### Heterogeneity across studies

Results varied depending on study design and type of depression outcome. Depression phenotyping varied substantially across studies, ranging from ICD-coded diagnoses and number of hospital admissions to symptom scales and antidepressant medication proxies, capturing overlapping but non-identical constructs. Studies using diagnosis-based outcomes, including ICD-based codes and clinical interviews, more often showed protective effects for ACEIs and ARBs, whereas those relying mainly on self-reported symptom scales tended to produce null findings. Diagnosis-based outcomes tend to prioritise specificity over sensitivity and may under-ascertain milder or untreated cases, whereas symptom scales capture a broader spectrum of distress and may be influenced by symptom burden. These sensitivity and specificity differences across outcome measures are likely to contribute to between-study heterogeneity and suggest that no single outcome definition should be treated as a gold standard across this literature.

Similarly, antidepressant prescribing is a flawed proxy for depression, considering antidepressants are frequently used for a range of mental health conditions [39, 40]. In addition, rather than disorder status, such prescription-based outcomes may capture treatment contact, prescribing preferences or healthcare access [40], increasing the risk of misclassifying depression status and biasing associations in either direction.

Differences in temporality also contributed to heterogeneity. Cohort studies using registry diagnoses generally assessed incident depression during follow-up, whereas cross-sectional studies using symptom scales captured prevalent depressive symptoms at a single timepoint. This distinction is important for interpretation, as prevalent symptom measures are more vulnerable to reverse causation and baseline differences in symptom burden.

Within-class pharmacological heterogeneity may also have contributed to inconsistency. ACEIs and ARBs differ in lipophilicity and blood-brain barrier penetration, yet most epidemiological studies classified exposure at the drug-class level and did not distinguish centrally penetrating from non-penetrating agents [41]. However, classification of central penetration is not always straightforward, and some agents traditionally considered to have limited blood-brain barrier penetration may still exert measurable central effects. Nevertheless, if central RAS effects are relevant to mood, combining such agents within the same exposure category could dilute true associations and increase between-study heterogeneity. Overall, interpretation should rest more heavily on studies with stronger confounding control than on the simple numerical balance of protective and null findings.

### Biological and mechanistic interpretation

Experimental research provides plausible biological mechanisms for a link between RAS modulation and mood regulation. Angiotensin II, acting at the AT1 receptor, promotes neuroinflammation and activation of the stress response, both implicated in depression [42, 43]. Blocking this pathway through ACEIs or ARBs reduces these effects in animal models and enhances neurogenesis and stress resilience [44, 45]. Further, AT1 receptors are present in dopaminergic pathways of the brain’s reward circuit [46], and have been shown to co-regulate dopaminergic neurotransmission [47]. This is of interest because impaired reward processing and reduced dopamine function are closely linked to anhedonia, an important dimension of depressive psychopathology [48], and a potential pathway through which RAS-modulating drugs may affect mood.

The AT4 receptor, expressed in brain regions relevant to cognition, has also been implicated in learning and memory processes [49]. Preclinical evidence suggests that some cognitive benefits attributed to losartan may partly depend on downstream angiotensin IV/AT4 receptor signalling rather than AT1 blockade alone [50]. Clinical studies in hypertension have reported improvements in cognitive measures and quality-of-life indices with losartan [51], which could plausibly influence self-report depressive symptom scales. These mechanistic observations may help explain heterogeneity across outcome types and agents, but they do not resolve the causal uncertainty of the observational literature. Further mechanistic work is needed to better characterise the mechanism through which RAS-modulating agents may influence mood in humans.

### Clinical implications

While these results are not sufficient to justify the use of RAS-modulating drugs as antidepressants, they strengthen the case for investigating the RAS pathway as a novel therapeutic target for depression, particularly in clinical subgroups characterised by high inflammation, anhedonia or reduced dopaminergic function. A deeper understanding of potential central neurobiological effects is therefore warranted.

Our findings are also relevant to clinical management of hypertension. Most participants in the included studies were adults treated for hypertension, a population with increased risk of depression. The frequent co-occurrence of hypertension and depression contributes to poorer cardiovascular outcomes, treatment non-adherence, and reduced quality of life [52]. Within this population, the absence of a consistent signal of increased depression risk with ACEIs or ARBs is clinically reassuring. However, current evidence is insufficient to recommend ACEIs or ARBs for prevention or treatment of depression.

### Limitations and directions for future research

All included studies were observational, and confounding by indication remains the central challenge to causal inference. ACEIs and ARBs are prescribed based on clinical characteristics such as cardiovascular disease severity, renal function, diabetes, and overall frailty, many of which are also associated with depression risk. As a result, differences in depression outcomes may reflect underlying patient characteristics rather than pharmacological effects. This could be seen in studies comparing treated and untreated groups, where baseline differences in health status and healthcare utilisation are difficult to fully account for. Although some studies used propensity scores or between-class comparisons, adjustment for key factors such as prior psychiatric history, socioeconomic position, and healthcare contact was inconsistent. Exposure misclassification was also possible when prescription records were used without information on adherence, dose, or duration, and outcome heterogeneity further limited direct comparison across studies.

Additional biases are also likely. Prevalent-user designs are vulnerable to survivorship bias, because long-term users represent a selected group who have tolerated and continued treatment, and healthy-user bias may arise if medication adherence tracks with broader health-promoting behaviours. Time-related biases may also occur when exposure classification and follow-up are not aligned. Future research should therefore prioritise bias-resistant pharmacoepidemiologic designs, particularly active-comparator new-user studies with clear temporal alignment between treatment initiation and incident depression outcomes. Studies should distinguish clinician-diagnosed incident depression from symptom-based outcomes, use drug-specific exposure definitions with information on dose, duration, adherence, and central penetration, and incorporate comprehensive adjustment for confounding. Extending analyses to populations without hypertension may also help clarify whether observed associations reflect direct central effects of RAS modulation or secondary consequences of cardiovascular disease. Prospective cohorts and pragmatic randomised comparisons, alongside translational studies integrating RAS-related biomarkers, inflammatory measures, reward processing, and neuroimaging, may further help identify subgroups in whom central RAS modulation is most relevant.

## Conclusion

Observational evidence suggests a modest protective association between ACEI or ARB exposure and depression outcomes, but findings are heterogeneous and less persuasive in studies with stronger control of confounding. More rigorous studies with incident depression phenotyping and interdisciplinary research integrating cardiology and psychiatry will be key to determining whether modulation of this pathway can meaningfully influence depression outcomes in clinical practice.
